# Moyamoya disease. An important differential diagnosis to cerebral vasculitis

**DOI:** 10.1186/1546-0096-9-S1-P85

**Published:** 2011-09-14

**Authors:** A Estmann, P Toftedal, AJ Schou

**Affiliations:** 1 Hans Christian Andersen Children’s Hospital, Odense, Denmark

## Background

Cerebral vasculitis is a serious but uncommon condition which presents considerable difficulties in recognition, diagnosis and treatment. Moyamoya disease is a progressive occlusive disease of the cerebral vasculature with particular involvement of the circle of Willis.

## Aim

To describe a case of moyamoya initial misinterpreted as cerebral vasculitis.

## Methods

A three-year-old girl, with a former history of Kawasaki disease, presented with a stroke. Both parents were of Korean heritage. Stenosis of the middle cerebral artery on both sides was seen on MR angiogram. No abnormal blood tests were found. Treatment of suspected cerebral vasculitis was initiated with steroids orally (2mg/kg/d). She developed recurrent transient ischemic attacks in both hemispheres and new strokes despite series of pulse therapy of steroids and Cyclophosphamide and finally Mycophenolic acid in combination with prednisolone daily. Stenosis of the anterior cerebral arteries developed and progressed and the middle cerebral arteries worsened despite treatment. Only minor changes were seen in the internal carotid arteries. Abnormal vascular collateral networks were only limited present.

Moyamoya was suspected and confirmed by conventional angiography and the child underwent surgical treatment. Superficial temporal artery to middle cerebral artery bypass ((STA-MCA-Bypass) and encephalomyosynangiosis was performed bilaterally. She recovered without any mental decline and is able to walk despite her hemi paresis.

## Conclusion

Moyamoya is a rare disease affecting the cerebral arteries, and an important differential diagnosis to cerebral vasculitis. Especially in the early course of moyamoya the radiological findings might be difficult to interpret.

